# Regulation of *Schistosoma mansoni* Development and Reproduction by the Mitogen-Activated Protein Kinase Signaling Pathway

**DOI:** 10.1371/journal.pntd.0002949

**Published:** 2014-06-19

**Authors:** Luiza Freire de Andrade, Marina de Moraes Mourão, Juliana Assis Geraldo, Fernanda Sales Coelho, Larissa Lopes Silva, Renata Heisler Neves, Angela Volpini, José Roberto Machado-Silva, Neusa Araujo, Rafael Nacif-Pimenta, Conor R. Caffrey, Guilherme Oliveira

**Affiliations:** 1 Grupo de Genômica e Biologia Computacional, Instituto Nacional de Ciência e Tecnologia em Doenças Tropicais and Centro de Excelência em Bioinformática- CEBio, Centro de Pesquisas René Rachou, Fundação Oswaldo Cruz – FIOCRUZ, Belo Horizonte, Minas Gerais, Brazil; 2 Biologia Parasitária, Departamento de Ensino, Pavilhão Arthur Neiva, Instituto Oswaldo Cruz, Fiocruz, Rio de Janeiro, Rio de Janeiro, Brazil; 3 Laboratório de Helmintologia Romero Lascasas Porto, Departamento de Patologia e Laboratórios, Faculdade de Ciências Médicas, Universidade do Estado do Rio de Janeiro, Rio de Janeiro, Rio de Janeiro, Brazil; 4 Laboratório de Esquistossomose Centro de Pesquisas René Rachou, Fundação Oswaldo Cruz - FIOCRUZ, Belo Horizonte, Minas Gerais, Brazil; 5 Laboratório de Entomologia Médica, Centro de Pesquisas René Rachou, Fundação Oswaldo Cruz - FIOCRUZ, Belo Horizonte, Minas Gerais, Brazil; 6 Center for Discovery and Innovation in Parasitic Diseases, California Institute for Quantitative Biosciences and the Department of Pathology, University of California, San Francisco, San Francisco, California, United States of America; University of Queensland, Australia

## Abstract

**Background:**

Protein kinases are proven targets for drug development with an increasing number of eukaryotic Protein Kinase (ePK) inhibitors now approved as drugs. Mitogen-activated protein kinase (MAPK) family members connect cell-surface receptors to regulatory targets within cells and influence a number of tissue-specific biological activities such as cell proliferation, differentiation and survival. However, the contributions of members of the MAPK pathway to schistosome development and survival are unclear.

**Methodology/Principal Findings:**

We employed RNA interference (RNAi) to elucidate the functional roles of five *S. mansoni* genes (SmCaMK2, SmJNK, SmERK1, SmERK2 and SmRas) involved in MAPK signaling pathway. Mice were injected with post-infective larvae (schistosomula) subsequent to RNAi and the development of adult worms observed. The data demonstrate that SmJNK participates in parasite maturation and survival of the parasites, whereas SmERK are involved in egg production as infected mice had significantly lower egg burdens with female worms presenting underdeveloped ovaries. Furthermore, it was shown that the c-fos transcription factor was overexpressed in parasites submitted to RNAi of SmERK1, SmJNK and SmCaMK2 indicating its putative involvement in gene regulation in this parasite's MAPK signaling cascade.

**Conclusions:**

We conclude that MAPKs proteins play important roles in the parasite *in vivo* survival, being essential for normal development and successful survival and reproduction of the schistosome parasite. Moreover SmERK and SmJNK are potential targets for drug development.

## Author Summary

Enzymes known as mitogen-activated protein kinases (MAP kinases/MAPKs) influence a number of essential biological activities, such as cell proliferation, differentiation and survival. However, for the *Schistosoma mansoni* flatworm parasite, very little is known about these enzymes. We used RNA interference (RNAi), a technique designed to decrease or stop the production of specific proteins of interest, to examine the contributions of five *Schistosoma mansoni* MAPKs to parasite growth and survival. After inducing the RNAi effect in young parasites, we then transferred the worms into mice and after 37 days, counted the number of surviving adult worms in the bloodstream, eggs in the liver, and examined those surviving worms for morphological defects. We found that RNAi of SmJNK decreases parasite survival by 56%, whereas RNAi of SmERK slows the maturation of the ovary and, thus, egg-laying. We also noted that c-fos, that is responsible for activating genes in the genome, was upregulated after RNAi of MAPKs. Our results help define the importance of MAPKs in the normal development and survival of the schistosome parasite and suggest one or more of these enzymes may be useful as drug targets to treat schistosomiasis.

## Introduction

Schistosomes are parasitic flatworms (Phylum Platyhelminths) that can survive for years or decades in the mammalian host [1], [2]. Besides strategies to inhibit or modulate host immune responses, the maintenance of homeostasis and complex cellular adaptations, *Schistosoma* integrates specific extracellular signals to generate an appropriate cellular response [3]. In this context, signal transduction has essential functions in the cell control involving non-linear integrated networks that interact mostly by switching the activity status of proteins.

The mitogen-activated protein kinase (MAP kinase/MAPK) signaling pathway is activated by a variety of extracellular growth factor-receptor interactions in response to environmental stimuli and leads to the downstream transcriptional activation of specific genes [4]. For example, in mammals, activated ERK MAPKs can translocate into the nucleus and induce phosphorylation of specific transcription factors such as ELK-1 [5]. ELK-1 forms a complex with another transcription factor, SRF (serum response factor), and the ELK-1/SRF complex is then able to bind to the promoter of the *c-fos* gene and trigger transcription [6]. MAPKs influence a number of tissue-specific biological activities like cell proliferation, survival and differentiation through the activation of other protein kinases, metabolic enzymes or by the phosphorylation of transcription factors and components of the cytoskeleton [7].

Recently we showed by *in silico* analyses that the MAPK signaling components are well conserved in the three main *Schistosoma* species that infect humans, namely *S. mansoni*, *S. japonicum* and *S. haematobium* [8]. These include representatives of the MAPK subfamilies ERK (extracellular signal-regulated kinase), p38, JNK (c-Jun N-terminal kinase) and nmo (nemo MAPK). However, a detailed understanding of MAPK pathway in schistosome development and survival remains to be elucidated.

In planarians, ERK plays a pivotal role in stem cell dynamics during regeneration. Activation of ERK signaling induces stem cells to exit proliferative state and enter the differentiating state [9]. In the *C. elegans* model nematode, ERK MAPKs are required for multiple developmental events, including the induction of vulval, uterine and spicule cell fates, and the promotion of germ line meiosis [10]. In *S. mansoni* Vicogne and colleagues (2004) [11] showed that the human epidermal growth factor (EGF) can activate the Ras/ERK pathway, which induces meiosis in oocytes. This is a relevant observation because oviposition is responsible for the pathogenesis of schistosomiasis. Females can release, on average, 300 highly immunoreactive eggs a day. Although, many eggs escape via body wastes, others become trapped in various tissues to elicit eosinophilic and granulomatous inflammatory reactions that give way to progressive fibrosis that can lead to organ dysfunction and, sometimes, death. These observations have led to our hypothesis that ERK MAPK pathway is involved in *Schistosoma* reproduction.

Apart from MAPKs, c-Jun N-terminal kinase (JNK) proteins also have evolutionary conserved functions, including the control of cellular responses to stress stimuli induced by a range of intrinsic and environmental aggression, e.g., UV irradiation, DNA damage, heat, bacterial antigens and inflammatory cytokines [12]. In addition, JNK signaling plays a crucial role during planarian regeneration by regulating the G2/M transition in the cell cycle of pluripotent stem cells [13]. In our *in silico* analyses, we showed that only one member of the MAPK JNK sub-family is encoded in the *S. mansoni* genome, in contrast to five genes expressed in *Caenorhabditis elegans* and three genes in humans [8]. This evolutionary constriction of the JNK subfamily in *S. mansoni* to just one enzyme suggests that SmJNK may be particularly worthy of investigation to understand its potential target for drug development as drug effectiveness can be marked when a single-copy gene is targeted [14].

The divalent cátion calcium (Ca^2+^) is one of the most widely ion used as a second messenger in cell signaling, and much of this process is controlled by calmodulin-binding kinase (CaMK) [15]. As *SmJNK*, only one *SmCaMK2* gene is encoded in the *S. mansoni* genome. In *C. elegans*, a JNK cell-specific pathway that is responsible for worm development, is activated by CaMK2 [10], [16]. Against this background, our study aimed at elucidating the function of ERK, JNK, CAMK2 and RAS, proteins involved in the MAPKs signaling pathways in the parasite *S. mansoni*, using RNA interference (RNAi). We show that RNAi of *SmERK* decreases egg production by female worms recovered from mice, which was consistent with the observations of an under-developed ovary and immature oocytes, and suggesting a direct involvement of SmERK in parasite reproduction. Furthermore, suppression of *SmJNK* gene expression killed the parasite and was associated with damage to the worm's tegument.

## Methods

### Ethics statement

Brazilian national guidelines set out in the Law 11794/08 were followed, stipulating the conditions for the use of animals in scientific research and setting up the National Council for the Control of Animal Experimentation (CONCEA) requiring the establishment of ethics committees on the use of animals (CEUA) by institutions under operational standards set out in Decree 6899/2009, including the principles of the Brazilian Society of Science in Laboratory Animals (SBCAL). Accordingly, animal experiments carried out in this work were approved by the Ethics Commission for Animal Use (CEUA) of Fundação Oswaldo Cruz under the number P49/12-5.

### Parasites

The LE strain of *Schistosoma mansoni* was maintained at Centro de Pesquisas René Rachou – FIOCRUZ using *Biomphalaria glabrata* as the intermediate snail host. Schistosomula were obtained by mechanical transformation of cercariae according to Howells *et al* (1974) [17] and cultured in MEM medium (*Minimum Essential Medium Eagle*) supplemented with 20 mM Hepes, 2 mM glutamate, 1×10^−6^ M serotonin, 5×10^−7^ M hypoxanthine, 2×10^−7^ M hydrocortisone, 0.5% MEM vitamin solution 100X, antibiotics (100 U/ml penicillin and 100 µg/ml streptomycin), and 2% fetal bovine serum (FBS).

### ERK/JNK phylogenetic analysis

In order to establish the evolutionary relationships among ERK and JNK proteins, homologs from *S. mansoni* (NCBI TaxID: 6183), *S. haematobium* (NCBI TaxID: 6185), *S. japonicum* (NCBI TaxID: 6182), *Caenorhabditis elegans* (NCBI taxID: 6239), *Drosophila melanogaster* (NCBI TaxID: 7227), and *Homo sapiens* (NCBI taxID: 9606) were selected for phylogenetic analysis. Amino acid sequences corresponding to the conserved catalytic domain (PF00069), present in JNK and ERK proteins, were aligned using MAFFT 7 with iterative refinement by the G-INS-i strategy [18] (Figure S1). The multiple sequence alignment comprising 34 sequences with 300 sites was manually refined using Jalview [19] and further used in phylogenetic analysis. To reconstruct the phylogenetic tree we used MrBayes (version 3.2.1), which performs Bayesian inference using a variant of the Markov chain Monte Carlo (MCMC) [20]. MCMC analyses were run as four chains for 10,000,000 generations and sampled every 100 generations. Of the initial samples, 25% were discarded as “burn-in.” Mixed models were applied as a parameter to estimate the best-fit evolutionary model. Support values were estimated as Bayesian posterior probabilities.

### Selected genes and primer design

The *S. mansoni* sequences were downloaded from *SchistoDB*, version 3.0 [21]. We selected five genes from the MAPK signaling pathway to perform the RNAi experiments: *SmCaMK2* (Smp_011660.2), *SmJNK* (Smp_172240), *SmERK1* (Smp_142050), *SmERK2* (Smp_047900) and *SmRas* (Smp_179910, previously characterized by [22]). In addition to the evaluation of transcript levels of those five genes, the transcription factors *SmSRF* (Smp_097730), *SmC-Fos*1 (Smp_124600) and *SmC-Fos*2 (Smp_170130) were also included in the analysis, in order to evaluate downstream interactions.

Procedures for dsRNA preparation, qPCR primer design and analysis, isolation of parasite RNA and reverse transcription to cDNA have been detailed previously [23] Primers were designed using the Primer 3 software (http://frodo.wi.mit.edu/) [24], [25] following strictly the MIQE guidelines [26] employing a 150–200 bp target product size for qPCR and 500–600 bp for templates of double stranded-RNA (dsRNA) (Figure S2). A T7 promoter tag was added to the 5′ end of all PCR primers designed for dsRNA template amplification (Table 1). A fragment of ∼500 bp of open reading frame for GFP (from the plasmid vector pCRII-GFP) was used as non- schistosome RNAi control [23]. Each qPCR primer was designed to anneal outside the targeted region of dsRNAs and was tested for primer annealing efficiency and optimal concentration. *SmCaMK2* (Smp_011660.1) has two predicted alternative splicing products (Smp_011660.2 and Smp_011660.3). The regions selected to design the dsRNA and to measure the transcription level by qPCR were identical in the three isoforms.

**Table 1:** Primer sequences.

	Gene ID	Primer name	Primer sequence
**DsRNA primers**	Smp_142050	SmERK1	Fow:5′-taatacgactcactatagggTTGGTCAATTGGTTGTATTATGG-3′
			Rev:5′-taatacgactcactatagggGGAACAATGGCACCAGGAAT-3′
	Smp_047900	SmERK2	Fow:5′-taatacgactcactatagggTCTGCCAGCGAACATATCG-3′
			Rev:5′-taatacgactcactatagggGGATCACCAAGTCGTGAAGA-3′
	Smp_011660.2	SmCaMK2	Fow:5′-taatacgactcactatagggGATGACATTCAGGACGAAGG-3′
			Rev:5′-taatacgactcactatagggTCGCAGGACTGACTGTTAG-3′
	Smp_172240	SmJNK	Fow:5′-taatacgactcactatagggACATGCAGCCGGTATAATCC-3′
			Rev:5′-taatacgactcactatagggTTACTTCAGAGTCTTCATACCATACG-3′
	Smp_179910	SmRas	Fow:5′-taatacgactcactatagggTGGCACCAGAACTTATCAGG-3′
			Rev:5′-taatacgactcactatagggGATATAGAGCAGTCATTGCATTCC-3′
	pCRII-GFP	GFP	Fow:5′-taatacgactcactatagggTCTTCAAGTCCGCCATG-3′
			Rev:5′-taatacgactcactatagggTGCTCAGGTAGTGGTTGTC-3′
**qPCR primers**	Smp_142050	qSmERK1	Fow:5′-TGCAACATCTTGTTGAATGC-3′
			Rev:5′-GCACGATACCAACGTGTACG-3′
	Smp_047900	qSmERK2	Fow:5′-TTATCCTTCGGCGGATGC-3′
			Rev:5′-AGCAACAGGCTCATCACTAGG-3′
	Smp_011660.2	qSmCaMK2	Fow:5′-ACGACTATGCTAGCCACACG-3′
			Rev:5′-CAGACGATTCCTTAATACCATCG-3′
	Smp_172240	qSmJNK	Fow:5′-TCCTCCTGGGTATCATGTCG-3′
			Rev:5′-GCTACAACAAAGCCCTGAGC-3′
	Smp_179910	qSmRas	Fow:5′-GACTGAGTACAAGTTAGTTGTTGTTGG-3′
			Rev:5′-TTCTATAAGAGTCCTCTATCGTTGG-3′
	Smp_124600	qSmc-Fos1	Fow:5′-GAGGCTGCAAGAGAATGTCG-3′
			Rev:5′-CAAAGTGCTTTAACTTTCTGAAGC-3′
	Smp_170130	qSmc-Fos2	Fow:5′-TTGTTTCTCGTCCATCCACA-3′
			Rev:5′-GAAACAGCTTGACGTTGTGC-3′
	Smp_097730	qSmSRF	Fow:5′-GATACCTATTGAATTTATTTCTGATCG-3′
			Rev:5′-CGGTTAATTCAGCCAATTCC-3′
	AF216698.1	COX	Fow:5′-TACGGTTGGTGGTGTCACAG-3′
			Rev:5′-ACGGCCATCACCATACTAGC-3′

### dsRNA synthesis and parasite exposure

Following amplification, PCR products were separated on 1% agarose gels and purified using QIAquick Gel Extraction Kit (QIAGEN). DsRNAs targeting specific *S. mansoni* genes were generated from PCR products of approximately 500 bp that had been amplified from schistosomula cDNA using the T7 RiboMAX Express RNAi Kit (Promega) as described elsewhere [23], [27]. Final dsRNA synthesis reactions were allowed to incubate for 16 h at 37°C prior to DNAse treatment. DsRNA was analyzed by electrophoresis in 1% agarose gels to ensure that the correct length of product was generated: sequence identity was confirmed by DNA Sanger sequencing.

Schistosomula (2,000 worms) were cultivated in 24-well polystyrene plates containing 2 mL MEM supplemented with 1% FBS, 100 U/ml penicillin and 100 µg/ml streptomycin. For each treatment, 100 nM of dsRNA were added in the first day. Incubations were carried for 2, 4 or 7 days at 37°C under 5% CO_2_. The experiments were performed in duplicate and in three biological replicates.

### Gene expression analyses (qPCR)

For qPCR, total RNA was extracted using the RNeasy Mini Kit (Qiagen). Residual DNA was removed by DNase digestion using the Turbo DNA-free kit (Ambion, Life Technologies). RNA (100 ng) was used to synthesize cDNA with the Superscript III cDNA Synthesis kit (Life Technologies). Each cDNA sample was tested in three technical replicates per plate using a minimum of 3 biological replicates. Experiments were carried out in a 7500 Real Time PCR System (Life Technologies) using the Power SYBR Green Master mix (Life Technologies). Reactions were carried out in a final volume of 25 µl in 96 well plates. *S. mansoni* cytochrome C oxidase I (GenBank AF216698) was used as the sample normalizing transcript [28], [29], as it has been shown to be highly and constitutively expressed in various *S. mansoni* life-cycle stages [30], [27] and GFP cDNA was used as endogenous control [31]. Two internal controls assessing both possible genomic DNA contaminations (no reverse transcriptase) and purity of the reagents (no cDNA) were included. The 2^−ΔΔCt^ method was used to measure transcript levels post-RNAi [32]. Transcript levels were expressed as percentage of difference compared to those following exposure to the schistosome- unspecific GFP dsRNA. Statistical analysis employed the Mann-Whitney *U*-test (p<0.05).

### *In vivo* experiments - adult worm and egg recovery

Swiss Webster mice were subcutaneously injected with 300 schistosomula 2 days after dsRNA treatment (3 independent experiments, 6 animals per group). After 37 days when the parasite has matured, mice were perfused according to Pellegrino and Siqueira (1956) [33] and the adult worms counted. Livers from infected animals were weighed and the eggs counted after digestion with 10% KOH. Statistical significance of the data was analyzed using the Mann-Whitney test (Wilcoxon-Sum of Ranks, p<0.05, N = 3).

Adult worm samples recovered after perfusion were analyzed by confocal microscopy. The parasites were fixed in AFA (2% acetic acid, 10% formaldehyde and 48% ethanol) and stored at room temperature. Whole worms were stained with 2.5% hydrochloric carmine, dehydrated by passage through 70, 90 and 100% ethanol, clarified with methyl salicylate and Canada balsam (1∶2), and individually mounted on glass slides.

Morphometric analyzes were performed on male and female worms using computer images (Image Pro Plus - Media Cybernetics, USA) captured by a Sony camera (640×480 pixels, RGB) coupled to a light microscope (Olympus BX50). The following parameters were determined: number and area of testicular lobes, area of the ovary, the presence of eggs and vitelline glands, the integrity of tegument and presence of surface tubercles. Statistical significance of the data was analyzed using the Mann-Whitney test (Wilcoxon-Sum of Ranks, p<0.05). It was analyzed 5 females that were treated with SmERK dsRNA, 6 for SmJNK, 8 for SMCaMK2 and 6 GFP control; 13 males that were treated with SmERK dsRNA, 5 for SmJNK, 6 for SmCaMK2 and 9 for GFP control.

Confocal microscopy images of the reproductive system and tegument were taken using a LSM-410, (Zeiss) equipped with a 488 nm HeNe laser and a LP 585 filter in reflected mode.

## Results

### ERK and JNK conservation

**Figure 1:**
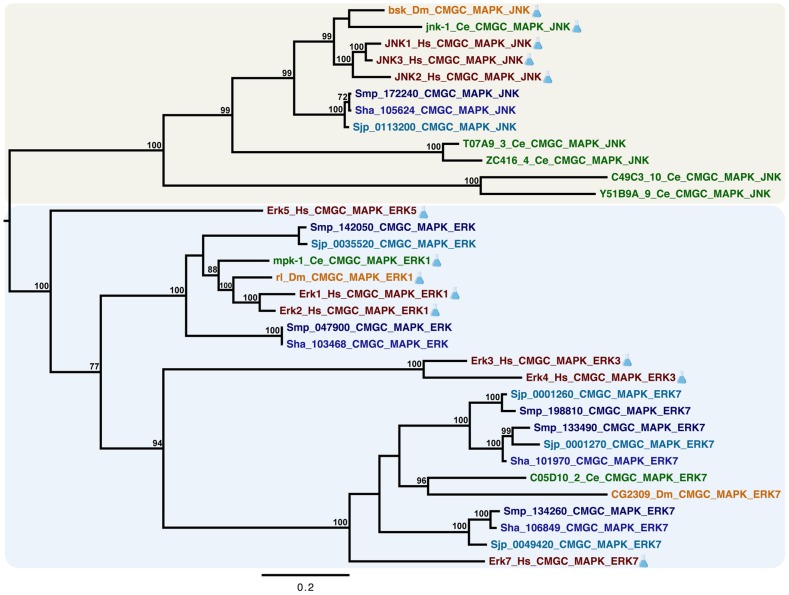
Evolutionary relationships of ERK and JNK proteins. Evolutionary relationships of 34 ERK and JNK proteins encoded by schistosome parasites (*S. haematobium*, *S. japonicum* and *S. mansoni* [different shades of blue]), *Caenorhabditis elegans* (green), *Drosophila melanogaster* (yellow), and *Homo sapiens* (red) as inferred by Bayesian analysis. Experimentally characterized proteins are indicated by an Erlenmeyer symbol. Different background colors highlight two clades: one containing ERK proteins and another containing JNK proteins. Support values were computed by posterior probability. The analysis was performed with conserved amino acid sequences corresponding to the catalytic domain (PF00069). Mixed models were selected as implemented in MrBayes with 10 million generations sampled every 100 generations.

It has previously been shown that *S. mansoni* expresses only one JNK sub-family member, which contrasts to the presence of five and three homologs in *C. elegans* and humans, respectively [8]. This evolutionary constriction of the JNK subfamily in *S. mansoni* suggests that the SmJNK protein may be a potential target for drug development. Aiming at characterizing the evolutionary relationships between ERK and JNK proteins encoded by parasites and free-living organisms, we performed phylogenetic analyses on selected homologs from three Platyhelminths (*S. mansoni*, *S. haematobium* and *S. japonicum*), one nematode (*Caenorhabditis elegans*), one arthropod (*Drosophila melanogaster*) and one chordate (*Homo sapiens*). This taxon sampling covers important evolutionary innovations in processes in which kinases are directly involved in responses to environmental stimuli such as reproduction and development. As shown by Figure 1, gene duplication followed by divergence was probably the main evolutionary mechanism driving the evolution of the ERK and JNK subfamily members. The tree topology shows two well-supported clades grouping ERK and JNK proteins, thus revealing that the catalytic domain (PF00069) is sufficiently divergent to discriminate these two protein subfamilies. The number of orthologs in schistosomes and other metazoans varies and the presence of sequence variants may implicate structural and/or functional specializations. In most cases, when orthologs were identified in the three *Schistosoma* species, the relationships among them reflected the current knowledge regarding the origin and evolution of the *Schistosoma* lineage [34]. Together, these findings demonstrate that ERK and JNK proteins are evolutionarily conserved in metazoan species transducing signals from the cell surface to the nucleus.

### MAPK members are susceptible to RNAi but knockdown efficiency varies

**Figure 2:**
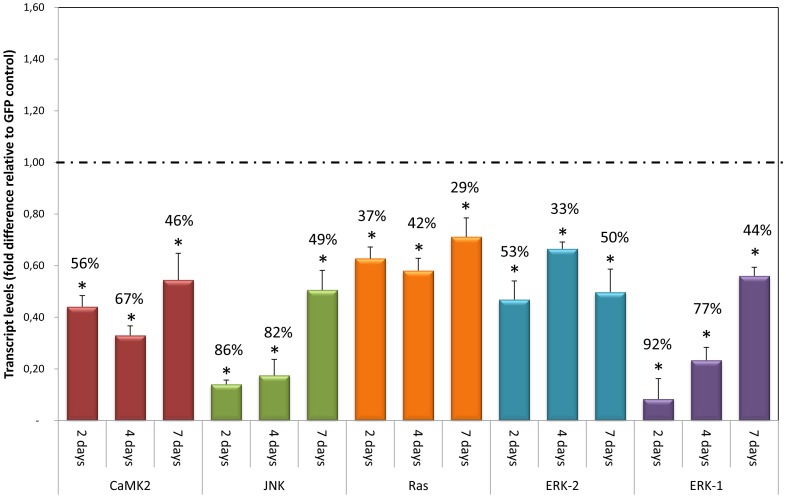
Transcript levels of target genes in schistosomula 2, 4, and 7 days after exposure to dsRNA. Bar graph indicating the relative steady-state transcript levels of SmCaMK2 (red), SmJNK (green), SmRas (orange), SmERK-2 (blue), and SmERK1 (purple) genes after 2, 4, and 7 days after dsRNA exposure. For each dsRNA tested, data are represented as mean fold-differences (+/−SE) relative to GFP control (1.00 – dashed line). Transcript levels were determined by qPCR and data analyzed using the ΔΔCt method [24], followed by statistical analysis using the Mann-Whitney U-test. Data were generated from 3 independent experiments, each one in duplicate, and all the data shown is statistically different from GFP controls.

To functionally characterize MAPK pathway members (SmERK1, SmERK2, SmJNK, SmCaMK2 and SmRas) by RNAi, we co-incubated schistosomula with synthetic double-strand RNAs (dsRNA) *in vitro*. Relative to schistosome-unspecific controls using dsRNA to GFP, all genes targeted were sensitive to RNAi and were substantially suppressed after two, four or seven days (Figure 2). Transcript levels were reduced by up to 92%, for *SmERK1*after two days (transcription levels relative to controls  = 0,08+/−0,0079) to 42% for *SmRas* after four days (transcription levels relative to controls  = 0,58+/−0,04) (Figure 2). Additionally, decreased transcript levels of *SmERK2* were observed in parasites two days after *SmERK1* dsRNA exposure (56% of inhibition: transcription levels relative to controls  = 0,44+/−0,006) (data not shown), this could be due to the similarity between *SmERK1* and *SmERK2*. After this observation we decided to call the ERK1 treatment ERK1/2.

### RNAi of SmJNK and SmERK limits parasite survival and/or fecundity *in vivo*

To investigate whether RNAi of *SmCaMK2*, *SmJNK* and *SmERK*1/2 impacts parasite viability *in vivo*, schistosomula were first incubated for two days with dsRNA and then transferred into mice (n = 6 per treatment). After 37 days, adult worms were perfused from the hepatic portal system and eggs recovered from livers. Due to the lack of effective RNAi knockdown, parasites treated with SmRas-dsRNA was not included in the *in vivo* test.

**Figure 3:**
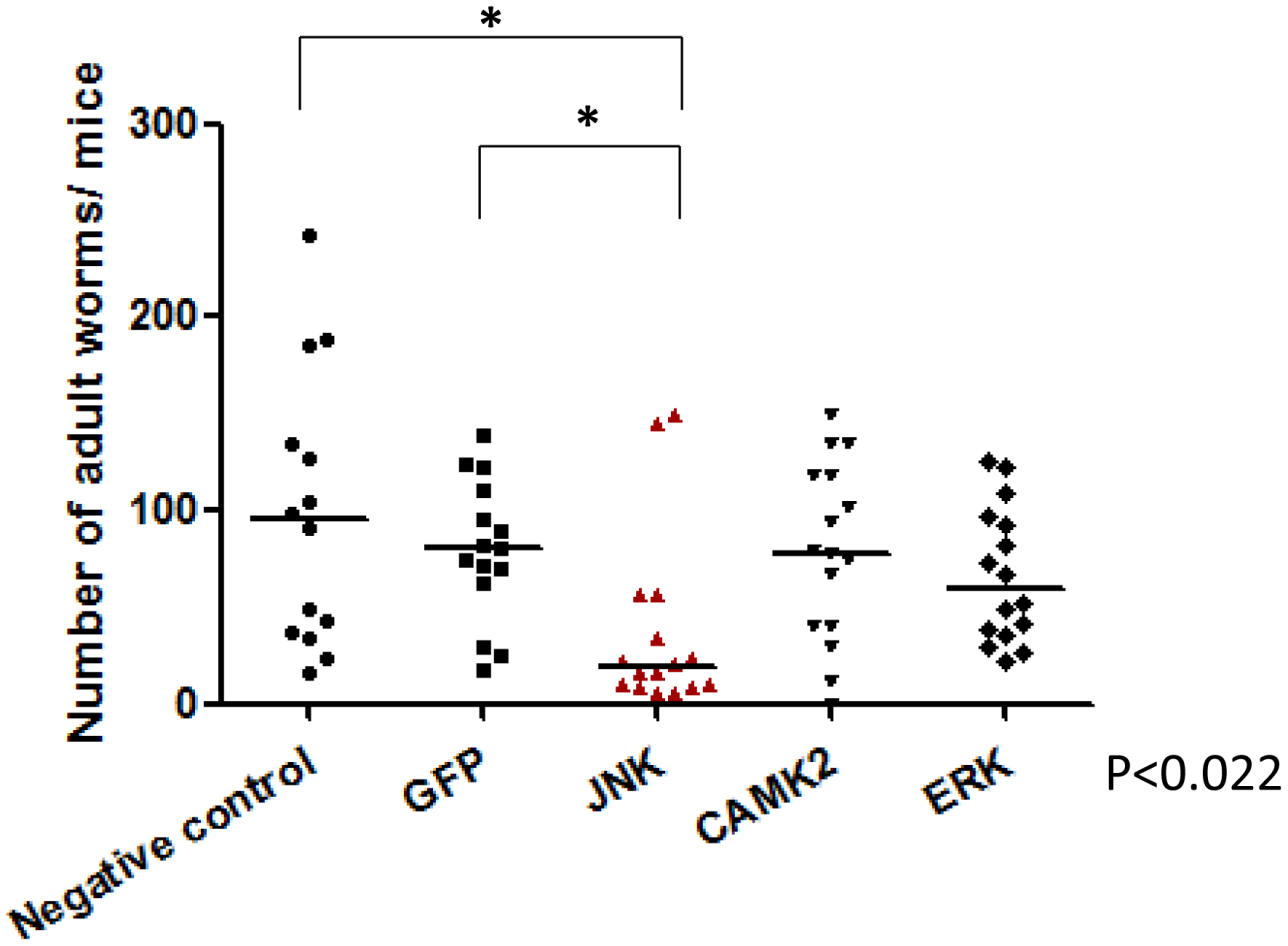
Survival of the parasite after RNAi of MAPKs *in vitro* and subsequent transfer into mice. Schistosomula were treated with GFP, SmJNK, SmCaMK2, and SmERK1 dsRNAs for two days and then injected into mice. After 37 days adult worms were recovered and counted. Each symbol in the chart represents worm counts from one mouse and the horizontal lines are median values per treatment group. Data were generated from 3 independent experiments and all treatments were statistical analyzed using Mann-Whitney U-test within each experiment (*P*≤0.05). The asterisk indicates a significance value of *P*<0.022 for RNAi of SmJNK relative to the GFP control.

**Figure 4:**
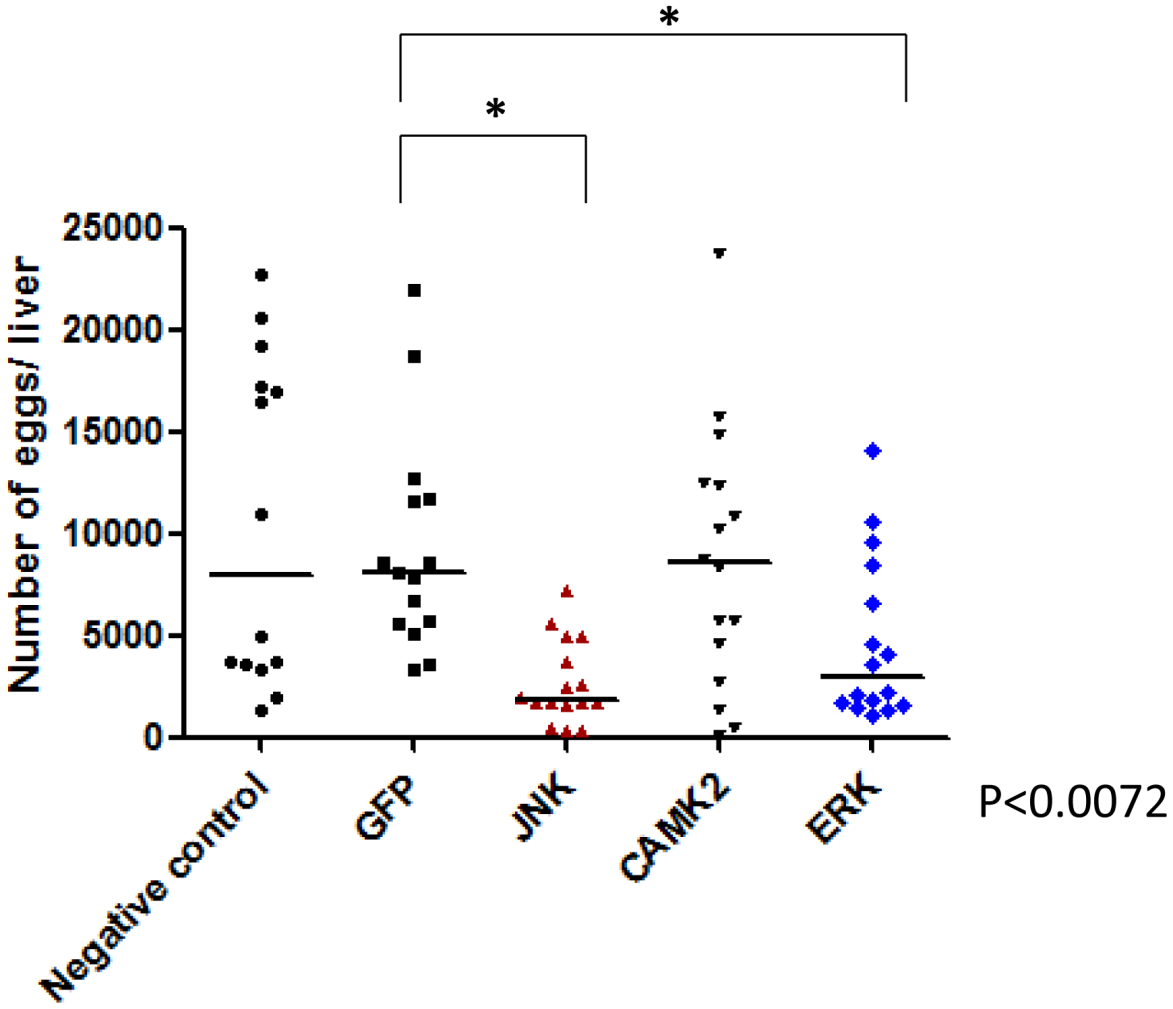
Hepatic egg counts after RNAi of MAPKs *in vitro* and subsequent transfer of parasites into mice. Schistosomula were treated with GFP, SmJNK, SmCaMK2 and SmERK1 dsRNAs for two days *in vitro* and then injected into mice. After 37 days of parasite eggs per liver digest were counted. Each symbol in the chart represents worm counts from one mouse and the horizontal lines are median values per treatment group. Data were generated from 3 independent experiments and all treatments were statistical analyzed using Mann-Whitney *U*-test within each experiment (P≤0.05). The asterisk indicates a significance value of SmJNK and SmERK *P*<0.0072 relative to the GFP control.

RNAi of *SmJNK* in schistosomula resulted in the death of 56% of parasites relative to the GFP control (Figure 3). Also, the number of hepatic eggs was decreased by 59% (Figure 4). For *SmCaMK2* and *SmERK1/2*, no significant changes in the number of adult worms were observed post-RNAi (Figure 3); also, RNAi of *CaMK2* did not alter egg output. On the other hand, although the knockdown of *SmERK*1/2 did not seem to affect parasite survival, egg production was decreased by 44% relative to parasites GFP-dsRNA treated (control) (Figure 4). Transcript levels of *SmCaMK2*, *SmJNK* and *SmERK*1/2 returned to its normal level of expression in 37 day-old worms (data not shown).

### RNAi of SmJNK and SmERK alters parasite morphology

**Figure 5:**
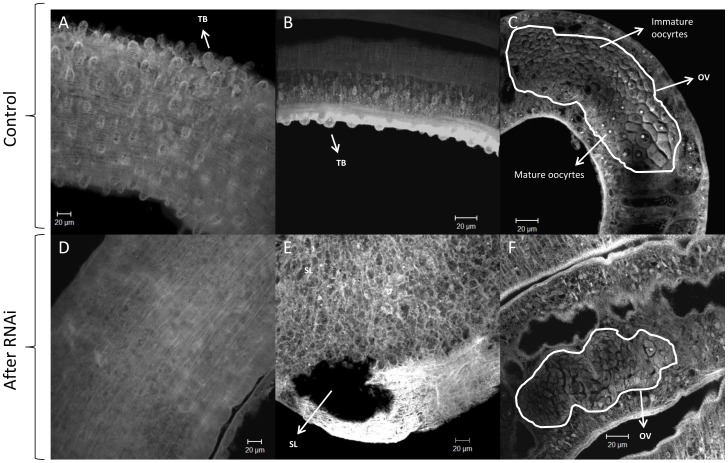
Morphology of adult worms after RNAi of SmJNK *in vitro* and subsequent transfer of parasites into mice. Adult 37-day-old worms were fixed and stained, and visualized by confocal microscopy as described in the text. A, B and C show normal worms treated with GFP dsRNA, whereas D, E and F show morphological changes in worms treated with SmJNK dsRNA. A and B - the tubercules (TB) are highlighted on the tegument; C – female worm ovary (OV) showing immature and mature oocytes; D – muscular structure of a worm without tubercules; E- subtegumentar lesion (SL); F- immature ovary.

Confocal microscopy was employed to understand whether parasite morphology was also altered in association with the decreased viability and/or egg production after RNAi of SmJNK and SmERK1/2. It was possible to observe that RNAi of SmJNK damaged the adult male tegument (Figure 5) in which the tubercles were reduced (Figure 5D) and unusual dilations were observed (Figure 5E). In addition, in the females control (Figure 5C) the ovary presents oocytes ranging from immature cells to mature cells with large and clearly nuclei and evident nucleolus, but the females worms treated with JNK dsRNA presented undifferentiated oocytes (cells throughout the uterus present the same size) (Figure 5F).

**Figure 6:**
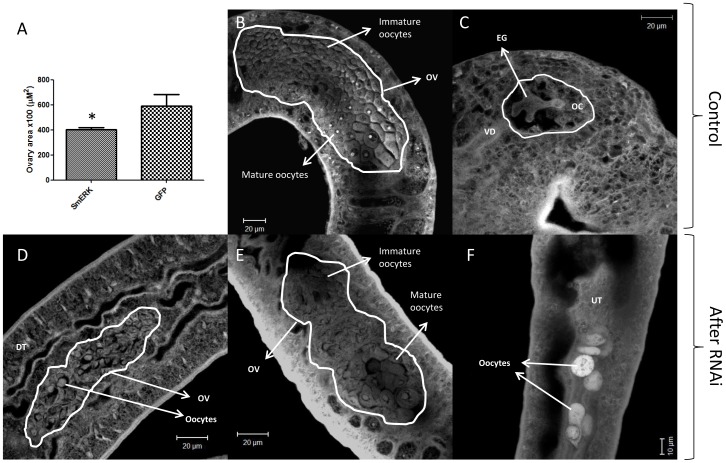
Morphology of adult female worms after RNAi of SmERK1/2 *in vitro* and subsequent transfer of parasites into mice. Adult 37-day-old worms were fixed and stained, and visualized by confocal microscopy as described in the text. A - mean of females' ovary area (µM^2^) of SmERK-knockdown and control showing a significant size reduction; B and C show normal worms treated with GFP dsRNA where the ovary (OV) with immature and mature oocytes, an egg (EG) and the vitelloduct (VD) are visible; D, E and F show morphological changes in worms treated with SmERK dsRNA where the ovary (D) present no mature oocytes (D) or even when mature oocytes are visible (E) an unexpected phenotype (a lot of oocytes) is observed in the uterus (UT) (F). The eggs shoud be fully formed in the uterus as showing in (C). Statistical analyses were performed using Mann-Whitney U-test, P≤0.05; n = 5).

The knockdown of SmERK1/2 did not cause changes in male worms (data not shown). The tegument and testicular lobes appeared to be normal and the seminal vesicle was full of spermatozoids. However, the females showed alterations in the reproductive system such as small ovaries (∼44% smaller than GFP controls) (Figure 6A) containing immature oocytes (Figure 6D) or, even when mature oocytes were observed (Figure 6E), a higher number of oocytes were present in the uterus (Figure 6F) whereas eggs were expected in this location, like the ones observed in the control group (Figure 6C).

In addition, RNAi of SmCaMK2 induced no apparent morphological alterations (Figure S3).

### RNAi of MAPK genes alters the transcript levels of downstream target genes

**Figure 7:**
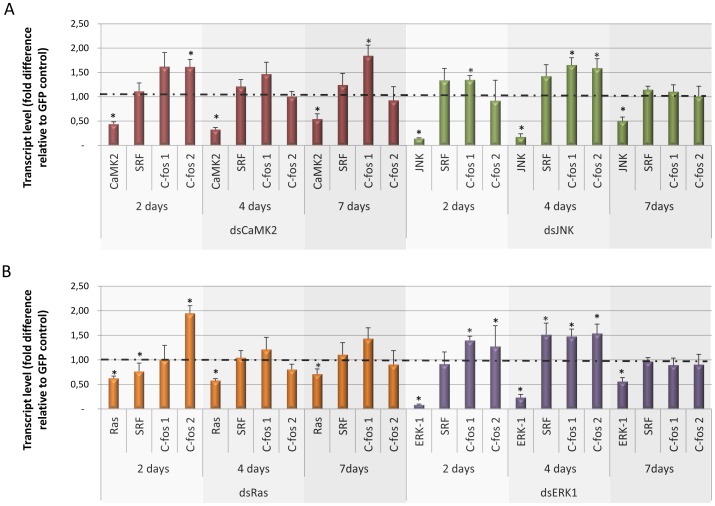
Transcript levels of *SmSRF* and *Smc-fos1* and *Smc-fos2* genes 2, 4, and 7 days after schistosomula were exposed to various MAPK dsRNAs. Bar graph indicating the relative steady-state transcript levels of (A) SmCaMK2 (red), SmJNK (green) and (B) SmRas (orange), SmERK1(purple) genes after 2, 4, and 7 days of dsRNA exposure. For each dsRNA tested, data are represented as mean fold-differences (+/−SE) relative to GFP control (1.00). Transcript levels were determined by qPCR and data analyzed using the ΔΔCt method [24] followed by statistical analysis using the Mann-Whitney *U*-test. Data were generated from 3 independent experiments, each one in duplicate. Significance levels (*) were set at *P*≤0.05.

In other organisms MAPK pathway, the downstream genes are transcribed when the ELK1/SRF complex binds to the promoter region of *c-fos* gene. To study the conservation of the MAPK pathway in *S. mansoni* compared to other metazoans, we measured the transcript levels of the SRF transcription factor and *c-fos* genes after RNAi of *SmCaMK2, SmJNK, SmRas* and *SmERK1*. RNAi of the first three targets caused the over expression of *Smc-fos1* (by 1.62+/−0.28; 1.65+/−0.14; 1.21+/−019 and 1.47+/−0.06, respectively) and *Smc-fos2* (by 1.65+/−0.15; 1.59+/−0.19; 1.95+/−0.24 and 1.53+/−0.02, respectively) (relative to the GFP control) (Figure 7). In addition, as the MAPKs (*SmJNK* and *SmERK-1*) transcript levels increased up to seven days, the *Smc-fos1 and Smc-fos2* RNA levels decreased (1.10+/−0.2 and 0.89+/−0.18) and (0.60+/−0.20 and 0.9+/−0.14), respectively (Figure 7). *SmSRF* gene expression, in most cases, did not exhibit variation. A minor alteration in *Smc-fos1* transcript level was observed after RNAi of *SmRas* (Figure 7B).

## Discussion

MAPKs connect cell-surface receptors to regulatory targets within cells to coordinate gene expression. Members of this family regulate essential cellular processes and are conserved in eukaryotes [4]. It would be expected that MAPKs also have important functions in the schistosome parasite, however, little was known. Here, we demonstrate RNAi for genes related to MAPK signaling pathway, namely: *SmJNK, SmERK-1, SmERK-2, SmCaMK2, SmRas*. Knockdown efficiency reached levels of up to 92% for *SmERK-1*, whereas *SmERK-2* was less susceptible with a 33% knockdown. Other authors [31], [27] also reported variable efficiencies of RNAi across different targets and for specific dsRNA sequences. It is possible that some genes are expressed in cells and tissues that are inaccessible to dsRNA and/or that the employed delivery method (soaking) did provide for maximal penetration of the RNAi effect. Also, the secondary structure of some mRNA targets might prevent or affect activation of the RISC complex [35].

Having demonstrated RNAi for the MAPKs studied, we next asked whether RNAi would limit survival and development of the parasites upon their transfer to mice. Thus, RNAi of *SmJNK* seems to be partially lethal and 56% of the parasites did not survive to 37 days, at which time worms were harvested from mice and counted. In addition, the recovered worms had morphological changes in the tegument. It's important to emphasize that survived worms may be affected to a lesser extent or maybe even not affected by RNAi treatment. Mourão and colleagues [31], after labeling the RNAi molecule with a fluorescent label, demonstrated that RNAi uptake was not equal among all parasites. So, the results suggest that SmJNK is an essential protein. This fact is reinforced by previous knowledge of JNK signaling pathway influencing metabolism, growth, regeneration, and stress tolerance in *Drosophila* lifespan regulation [36]. Moreover, in flies, the JNK signaling pathway is also involved in midgut epithelial homeostasis and may be important in other contexts, such as oxidative stress for protection against gut infections [37]. A strong inhibition of JNK signaling activity in *Drosophila* shortens lifespan due to complete inhibition of intestinal stem cells proliferation [36]. JNK signaling misregulation has also been implicated in regeneration, neurodegenerative diseases, diabetes, and cancer [38], [39], [40], [41], [13]. Moreover, JNK is only encoded by one gene in *Schistosoma* and *Drosophila* which is in contrast to the five subfamily members in *C. elegans* and three in humans. Accordingly, it's conceivable pivotal importance in the MAPK pathway and for downstream signaling may prove a valuable target point for small molecule interventions.

In *C. elegans*, the JNK pathway is also activated by CaMK (unc-43, a calcium/calmodulin-dependent protein kinase) in a cell-specific signaling pathway [10], [16]. As JNK, only one CaMK2 protein was found in the predicted proteomes of *S. mansoni* [42], [8] and *S. haematobium*. Although *SmCaMK2* has been predicted to be an essential gene and potential drug target [42], our present findings showed that RNAi of *SmCaMK2* does not alter worm morphology or survival in mice. A simple explanation is that RNAi of *CAMK2* was not efficient enough (ranged between 46 and 67%) to generate a phenotypic outcome. We also do not exclude the possibility that CaMK2 may regulate the JNK pathway only in particular cell type(s) or that the CaMK2 protein turnover is faster than that of SmJNK. Although, the same phenotype for SmJNK and SmCaMK2 was expected, since CaMK2 (UNC-53) can activate JNK signaling pathway in *C. elegans*, no alteration was observed after CaMK2 dsRNA treatment in *S. mansoni*. There are at least two possible explanations to this outcome: i) SmCaMK2 is not related to JNK signaling pathway or ii) SmCaMK2 is not the only activator of JNK signaling pathway in *Schistosoma*.

RNAi of *SmERK*1/2 decreased the number of parasite eggs recovered from the liver and apparently elicited morphological alterations only in the reproductive system of female worms. Our data are consistent with the known contributions of ERK to oocyte maturation and egg activation in other animals [43], [44]. Thus, in *Xenopus laevis*, the ERK protein is involved in the coordination of oocyte maturation [45]. In C. elegans, the knockout of *ERK*, affects the development of the vulva (necessary for egg-laying) and oocytes, resulting in a loss of egg production [46]. In a closely related organism, *Echinococcus multilocularis* it has been demonstrated that the Erk-like MAPK is activated by soluble host growth factors that are released by host hepatocytes and triggers metacestode development in vitro [47]. Moreover, the use of Erk-like MAPK pathway inhibitors affected *E. multilucularis* development and growth, but did not induce mortality [48]. In mice, the inactivation of the ERK signaling pathway is associated with embryonic death caused by abnormal placental development [49]. Sandler and colleagues also demonstrated that ERK is involved in starfish egg apoptosis [50]. These data are consistent with the current results and suggest a functional conservation of the ERK pathway across metazoans.

The effects observed after the knockdown of *SmERK*1/2 and *SmJNK* MAPKs were probably the consequence of gene transcription modulation that occur downstream of the MAPK signaling pathway. In other systems, SRF is a transcription factor known to regulate the transcription of the *c-fos* gene after MAPK activation [6]. For examples, in mammals, the *c-fos* gene has a variable level of transcription that is dependent on elk-1/SRF binding. The latter complex, in turn, has a less variable transcription rate as it presents a stable conformation in either active (On) or inactive (Off) modes [51]. In order to determine whether the MAPK pathway induces *c-fos* expression in *S. mansoni*, c-fos and SRF transcripts levels were evaluated after transcript knockdown of *SmRas*, *SmERK*1/2, *SmJNK* and *SmCaMK2*.

In general, we noted that the transcript levels of *SmSRF* remained constant. However, it was noted a different regulation (up or down) of *SmSRF* after SmRAS and SmERK dsRNA treatment (Figure 7) that may be an influence of some negative feedback regulation, a wide-spread mechanism among signaling molecules, especially in the MAPK pathway [52], [53]. On the other hand, the transcription of *Smc-fos1* and *Smc-fos2* is upregulated after RNAi of MAPK pathway genes. Thus, SmERK1/2, SmCaMK2 and SmJNK negatively regulated *c-fos*, whereby low levels of those proteins induce *c-fos* transcription. This contrasts with mammalian systems in which the inactivation of ERK and JNK prevents SRF activation, which, in turn, does not bind to the *c-fos* promoter region [5].

In contrast to the mammalian c-fos activation mechanism, *C. elegans* has a pathway that is consistent with our results observed for *S. mansoni*. Specifically, elk-1 (LIN-31 in *C. elegans*) and SRF (LIN-1 in *C. elegans*) form a complex when MAPKs are not phosphorylated that activates c-fos, which then inhibits vulval development. When MAPK is phosphorylated, the elk-1/SRF complex dissociates and elk-1 promotes vulval development in a signaling pathway that is activated by epidermal growth factor (EGF) [54]. Moreover, it was recently reported that pathways involved in activating c-fos gene expression might be themselves activated by calcium influx through the CaMK signaling pathway [50]. In this case, c-fos expression is induced by phosphorylation of CaMK2 which, in turn, phosphorylates SRF without a direct relationship to ERK or JNK proteins [55].

**Figure 8:**
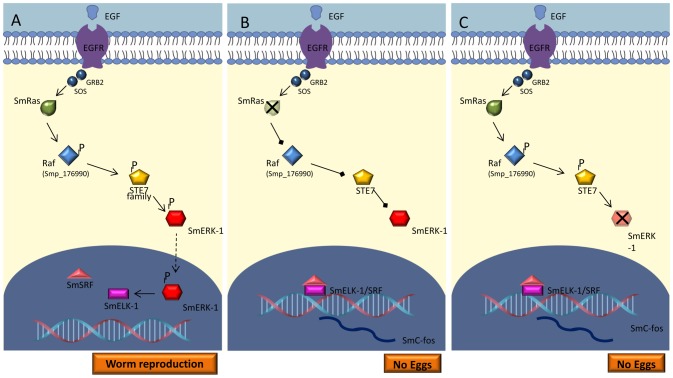
Hypothetical *S. mansoni* MAPK signaling pathway. (A) EGF activates the Ras/ERK signaling pathway. EGFR transmits the signal to the intracellular environment through the activation of Ras and sequential phosphorylation of SmRAF (ePK of TKL group and raf family), SmSTE7 (ePK of STE group and STE7family) and SmERK (ePK of CMGC group, MAPK family and ERK1/2 subfamily). Activated ERK translocates to the nucleus and inhibits the formation of the elk1/SRF complex and, in this case, oviposition remains constant. In B and C, SmRas or SmERK activity is interfered with (via RNAi) and the signal is not transmitted. Elk1 forms a complex with SRF which binds to the c-Fos promoter and this initiates c-Fos transcription that subsequently prevents the egg laying.

Together, it is possible that the high levels of *Smc-fos1* and *Smc-fos2* transcripts, as a result of RNAi of *SmERK*, *SmJNK* and *SmCaMK2*, is related to the inactivation of the MAPK signaling pathway which then induces the formation of the elk-1/SRF complex (Figure 8B–C). The elk-1/SRF complex is targeted by different signaling cascades and is involved in the regulation of *c-fos*. In *S. mansoni*, even though the outcomes of RNAi of *SmJNK* and *SmCaMK2* were quite different, it does seem that both gene products contribute to the regulation of the *c-fos* gene. SmJNK and SmCaMK2 may be involved in independent pathways or they may simultaneously co-regulate the same gene in a particular cell type. On the other hand, SmRas and SmERK would act in the same pathway, as is the case for *C. elegans*, mammals and *Drosophila*, being directly involved in the development of *S. mansoni* eggs (Figure 8A).

Our findings using RNAi demonstrate that *S. mansoni* MAPKs are essential to worm survival and/or reproduction suggesting that one or more of these kinases may be of interest in the development of new compounds to treat schistosomiasis. The complete mechanism by which MAPKs regulate those systems in *Schistosoma* still have to be elucidated to better focus on the most promising drug target.

Accession number for SchitoDB [21]: SmCaMK2 (Smp_011660.2), SmJNK (Smp_172240), SmERK1 (Smp_142050), SmERK2 (Smp_047900), SmRas (Smp_179910), SmSRF (Smp_097730), SmC-Fos (Smp_124600), SmC-Fos2 (Smp_170130).

## Supporting Information

Figure S1 **Multiple sequence alignment of ERK and JNK proteins encoded by parasites and free-living organisms.** Amino acid sequences of the conserved catalytic domain (PF00069) were aligned using MAFFT 7 with iterative refinement by the G-INS-i strategy [18]. The multiple sequence alignment comprising 34 sequences with 300 sites was manually refined using Jalview [19]. The most conserved and important aminoacids for the catalitic activity are highlighted in the aligment (A–E).
(TIF)

Figure S2 ***S. mansoni*** **dsRNA primers location.** Protein ID is shown above each image. The total length of each gene and the DsRNA forward and reverse primer position are represented in the figure.
(TIF)

Figure S3 **Morphology of adult male and female worms after RNAi of SmCaMK2** ***in vitro*** **and subsequent transfer of parasites into mice.** Adult 37-day-old worms were fixed and stained, and visualized by confocal microscopy as described in the text. A, C and E show male worms, whereas B, D and F shows female worms treated with SmCaMK2 dsRNA. No alterations are visible. It is possible to see that the testicular lobes (TL) are normal (A), seminal vesicle (SV), the duct for seminal pore (DP) and genital pore are visible (C), and tubercles (TB) are present in the tegument (E). The egg (EG) is fully formed (B), the ovary (OV) present mature and immature oocytes (D) and spermatozoides are visible in the spermathec (ES) (F).GP: genital pore; DP: duct for seminal pore; VD; vitelloduct; DT: digestive tract.
(TIF)
