# Correction: Regulation of *Schistosoma mansoni* Development and Reproduction by the Mitogen-Activated Protein Kinase Signaling Pathway

**DOI:** 10.1371/journal.pntd.0003081

**Published:** 2014-07-14

**Authors:** The PLOS Neglected Tropical Diseases Staff

## Notice of Republication

This article was republished on June 25, 2014, to correct errors in the figures that were introduced during the production process. Please download this article again to view the correct version. The originally published, uncorrected article and the republished, corrected article are provided here for reference.

## Supporting Information

File S1 Originally published, uncorrected article.
(PDF)

File S2 Republished corrected article.
(PDF)
