# Supplementary figures and images for: Regulation of Schistosoma mansoni Development and Reproduction by the Mitogen-Activated Protein Kinase Signaling Pathway
Source: PLoS Negl Trop Dis. 2014 Jun 19;8(6):e2949. doi: 10.1371/journal.pntd.0002949 (PMC4063740; doi:10.1371/journal.pntd.0002949)

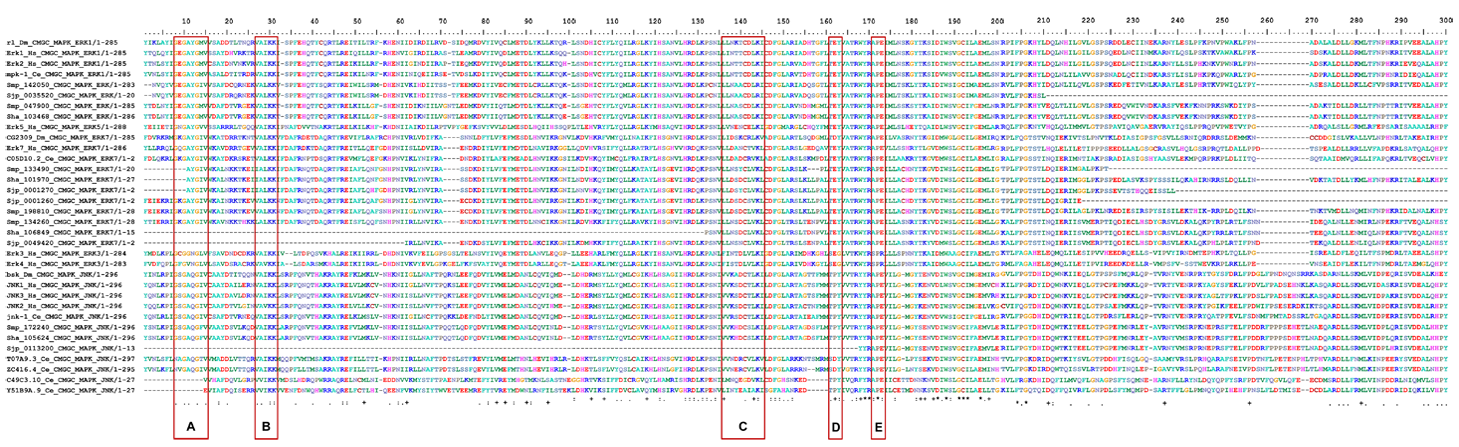

Supplement: Figure S1 — Multiple sequence alignment of ERK and JNK proteins encoded by parasites and free-living organisms. Amino acid sequences of the conserved catalytic domain (PF00069) were aligned using MAFFT 7 with iterative refinement by the G-INS-i strategy [18]. The multiple sequence alignment comprising 34 sequences with 300 sites was manually refined using Jalview [19]. The most conserved and important aminoacids for the catalitic activity are highlighted in the aligment (A–E). (TIF) [file pntd.0002949.s001.tif]

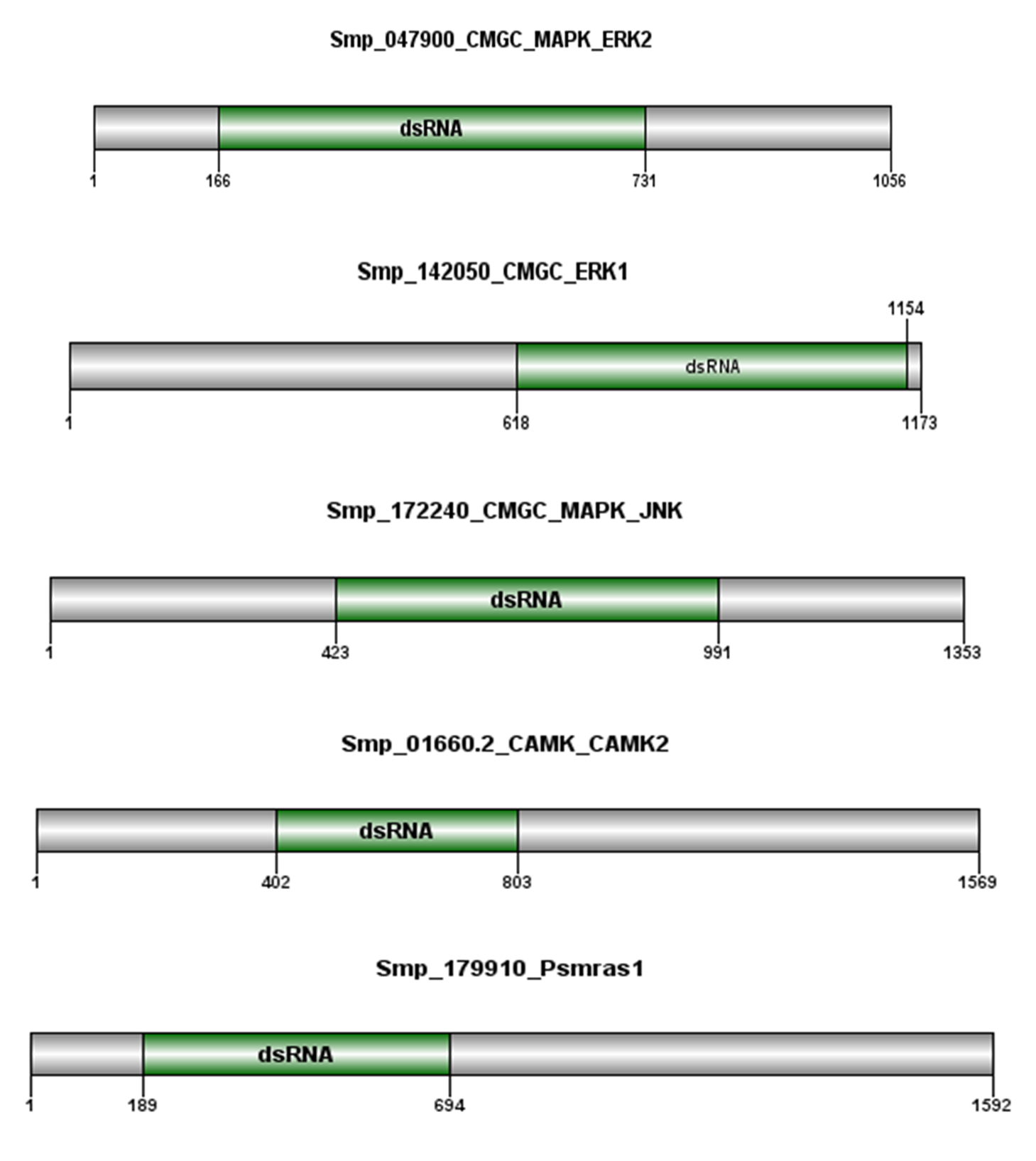

Supplement: Figure S2 — S. mansoni dsRNA primers location. Protein ID is shown above each image. The total length of each gene and the DsRNA forward and reverse primer position are represented in the figure. (TIF) [file pntd.0002949.s002.tif]

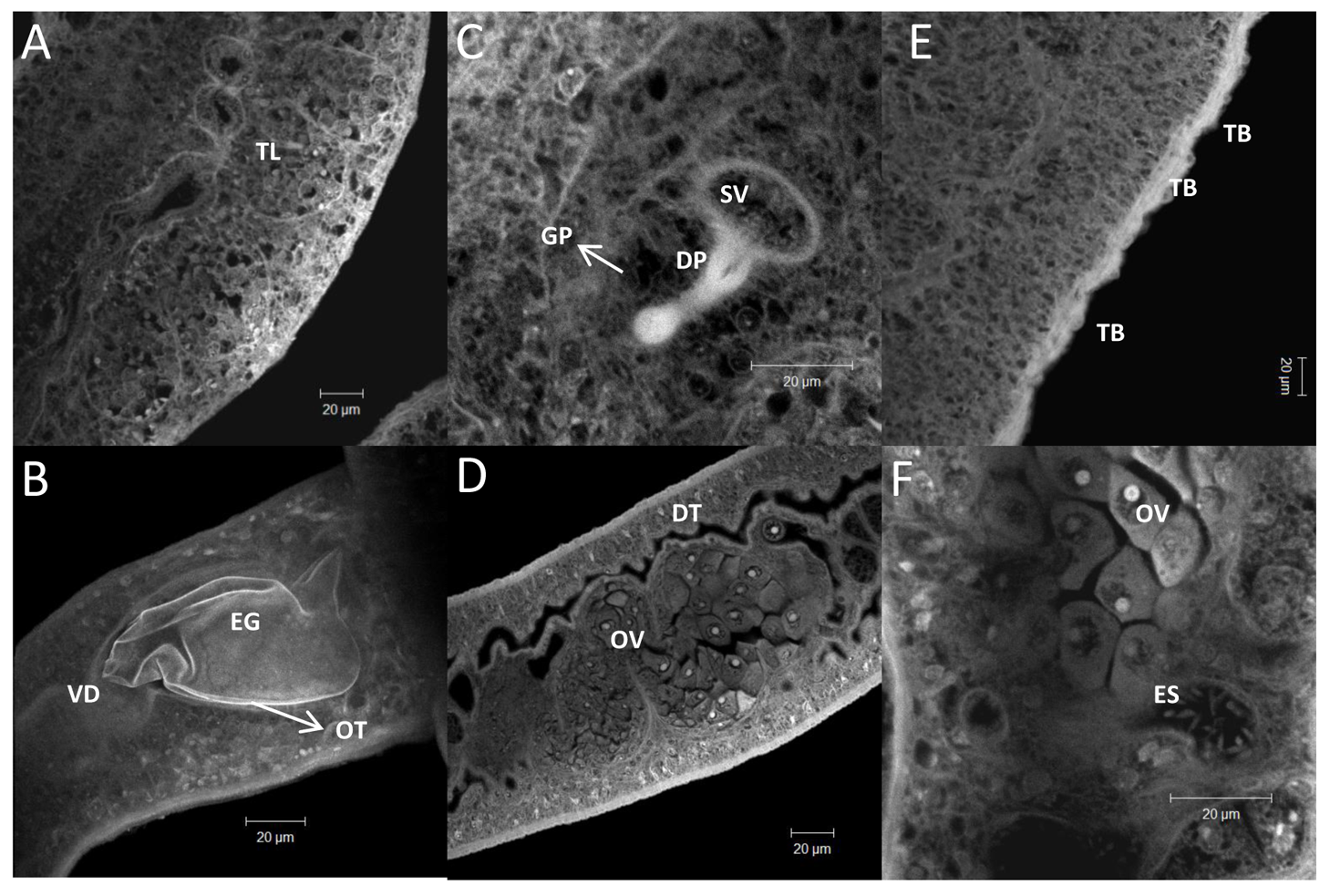

Supplement: Figure S3 — Morphology of adult male and female worms after RNAi of SmCaMK2 in vitro and subsequent transfer of parasites into mice. Adult 37-day-old worms were fixed and stained, and visualized by confocal microscopy as described in the text. A, C and E show male worms, whereas B, D and F shows female worms treated with SmCaMK2 dsRNA. No alterations are visible. It is possible to see that the testicular lobes (TL) are normal (A), seminal vesicle (SV), the duct for seminal pore (DP) and genital pore are visible (C), and tubercles (TB) are present in the tegument (E). The egg (EG) is fully formed (B), the ovary (OV) present mature and immature oocytes (D) and spermatozoides are visible in the spermathec (ES) (F).GP: genital pore; DP: duct for seminal pore; VD; vitelloduct; DT: digestive tract. (TIF) [file pntd.0002949.s003.tif]
